# Editorial Comment: Parasacral transcutaneous electrical nerve stimulation in children with overactive bladder: comparison between sessions administered two and three times weekly

**DOI:** 10.1590/S1677-5538.IBJU.2020.0372.1

**Published:** 2021-07-20

**Authors:** José Ailton Fernandes Silva

**Affiliations:** 1 Universidade do Estado do Rio de Janeiro Disciplina de Urologia Núcleo Disfunções Miccionais Rio de JaneiroRJ Brasil Disciplina de Urologia Núcleo Disfunções Miccionais, Universidade do Estado do Rio de Janeiro - PPC-UERJ, Rio de Janeiro, RJ, Brasil

## COMMENT

The parasacral TENS has been used to treat OAB in children however, the periodicity with which it should be carried out has not been yet standardized. This is a randomized clinical trial designed to compare two and three times weekly TENS (20 sessions of 20 min each at 10 Hz.) in children with OAB. The study included children of at least four years of age with a diagnosis of pure OAB based on ICCS definition (1). All the children were treatment naive and also underwent simultaneous standard urotherapy with TENS.

Sixteen children were included in the twice-weekly group and eighteen in the three times weekly group (2). There were no statistically significant differences regarding complete resolution of urinary symptoms, as evaluated by the visual analogue scale, with 8 children (50%) in the twice-weekly group and 11 children (61%) in the three times weekly group having their symptoms completely resolved. There was a significant difference in the DVSS score in both groups following TENS treatment compared to baseline.

Both groups experienced an improvement in symptoms of urgency and urge incontinence. Evaluation of the bladder diary showed no difference in mean urinary frequency, mean urine volume voided or maximum urine volume voided between the two groups.

Based on the results, the study suggested that it is possible to administer parasacral TENS twice a week, since there was a significant reduction in lower urinary tract symptoms in both groups.

Parasacral TENS has emerged as an effective non invasive treatment for OAB in view of its success rates in improving lower urinary tract symptoms without direct side effects. However, periodicity and comparison between applications conducted at home or an outpatient basis have not been yet defined. Considering that issues of cost effectiveness and adherence must be considered and put on the table in light of the evidence, more randomized clinical trials with a larger number of patients are necessary for better standardization of the technique.
